# Data on comparative proteomic profiling of human sperm affected by 4-tert-octylphenol in vitro

**DOI:** 10.1016/j.dib.2018.11.069

**Published:** 2018-11-16

**Authors:** Shaoping Huang, Senyang Cao, Tao Zhou, Lu Kong, Geyu Liang

**Affiliations:** a Department of Histology and Embryology, Medical School, Southeast University, Nanjing 210009, Jiangsu, China; b State Key Laboratory of Reproductive Medicine, Nanjing Medical University, Nanjing 210029, Jiangsu, China; c Center of Reproductive Medicine, Yancheng Maternity and Child Health Care Hospital, Yancheng 224002, Jiangsu, China; d Central Laboratory, Wuxi Maternity and Child Health Care Hospital Affiliated to Nanjing Medical University, 214002 Jiangsu, China; e School of Public Health, Southeast University, Nanjing 210009, Jiangsu, China

## Abstract

This data article presents proteomic profiling and posttranslational modifications of sperm proteins relating to the research paper “4-tert-octylphenol injures motility and viability of human sperm by affecting cAMP-PKA/PKC-tyrosine phosphorylation signals.” (Huang et al., 2018). Comparative proteomics was applied to identify the target biomarkers and the relevant molecular events of human sperm which were exposed to 0 (Dimethyl sulfoxide, DMSO), 0.1, or 0.3 mM 4-tert-octylphenol (4t-OP) for two hours in vitro. All differentially expressed (DE) proteins were then mapped to the human sperm proteome 2.0 (Wang et al., 2016) to clarify the posttranslational modifications (PTMs) of the DE proteins. We provide the associated mass spectrometry raw files; the PTMs in all the DE proteins, in DE proteins related to apoptosis, and in DE proteins related to motility. These data highlight the molecular mechanism relating to injured motility and viability of human sperm affected by 4t-OP.

**Specifications table**

**Table:** 

Subject area	Reproduction
More specific subject area	Xenoestrogen, male reproduction, sperm
Type of data	Table
How data was acquired	Mass spectroscopy and human sperm proteome 2.0
Data format	Raw, analyzed
Experimental factors	Healthy human sperm were exposed to 0, 0.1, or 0.3 mM 4t-OP for 2 h in vitro
Experimental features	Comparative proteomic profiling of human sperm exposed to 0, 0.1, or 0.3 mM 4t-OP; and PTMs of all the DE proteins.
Data source location	Department of Histology and Embryology, Medical School, Southeast University, Nanjing, China.
Data accessibility	Data is provided within this article
Related research article	Huang S, et al., 4-tert-octylphenol injures motility and viability of human sperm by affecting cAMP-PKA/PKC-tyrosine phosphorylation signals, Environ. Toxicol. Pharmacol. 62 (2018) 234–243 [1].

**Value of the data**

• Proteomic profiling presents potential biomarkers related to injured motility and viability of human sperm affected by 4t-OP, that could be used in researches of male fertility impacted by environmental polluters.
• Analysis of PTMs reveals that the major PTMs of DE proteins affected by 4t-OP, that would be valuable for studying posttranslational modifications of sperm proteins.
• These data provide an insight into the molecular mechanism related to impaired motility and viability of sperm caused by 4t-OP.

## 1 Data

The present data provide the mass spectrometry raw files and PTMs of the DE proteins associated to human sperm exposed to 0 (DMSO), 0.1, or 0.3 mM 4t-OP for two hours in vitro. Our proteomic files identified 111 protein spots with differential expression, corresponding to 81 human sperm proteins (see Supplementary Table 1). Analyzed with the human sperm proteome 2.0 [2], data presented the PTMs in all the DE proteins, in DE proteins related to apoptosis, and in DE proteins related to motility (see Supplementary Table 2).

## 2 Experimental design, materials and methods

### 2.1 Preparation of human sperm

The study was permitted by the Ethical Committee (study number: 20090598), Nanjing Medical University, Nanjing, China. As indicated in our previous paper [3], semen donors were informed with consent at the Reproductive Medicine Center, First Affiliated Hospital of Nanjing Medical University. Qualified semen samples (grade *a* + *b* > 50% and grade *a* > 25%), according to World Health Organization׳s criteria (1999), were used in this research. Purified sperm was obtained by removing the seminal plasma, somatic cells, and immature spermatogenic cells after centrifugation; then, sperm were cultured in human tubal fluid (HTF; in vitro Care, Frederick, MD, USA), concentration of sperm was 5.0 × 10^6^ cells∙ml^-1^.

### 2.2 Exposure of sperm to 4t-OP

As indicated in our research paper [1], DMSO (Sigma, St. Louis, USA) was applied as solvent; 400 mM stock solution of 4t-OP was prepared (Chem Service, West Chester, PA, USA) and stored in −26 °C. In present experiment, terminal concentration of DMSO in HTF was <0.1% for avoiding the impacts of DMSO on sperm. After exposure to 0 (DMSO), 0.1, 0.3 mM 4t-OP at 37 °C with 5% CO_2_ for 2 h, sperm were collected for further analysis.

### 2.3 Protein extraction

As indicated in our previous paper on mass spectrometry [3], sperm were collected and washed by phosphate-buffered saline (PBS, pH 7.2), centrifuged at 2000*g* for 5 min, three times. Sperm samples were incubated with lysis buffer containing 1% protease inhibitor cocktail (w/v) (Pierce Biotechnology, Rockford, IL, USA) in 4 °C for 1 h. Protein lysate was collected by discarding the insoluble material after centrifugation at 40,000*g*, 4 °C for 1 h. Protein concentration was detected by Bradford method using the bovine serum albumin (BSA) as an internal standard [4].

### 2.4 Two-dimensional electrophoresis (2-DE)

As indicated in our previous paper on mass spectrometry [4], 180 μg sperm protein lysate of each individual was loaded on immobilized pH gradient (IPG) strips (Amersham Bioscience, Uppsala, Sweden). In all, 9 IPG strips from three groups (0, 0.1 and 0.3 mM 4t-OP) were isoelectric focused and equilibrated, then electrophoresed in an Ettan DALT Twelve Electrophoresis System (GE Healthcare, San Francisco, CA, USA). The 9 gels were stained by silver nitrate dye; protein spots were analyzed by ImageMaster^TM^ 2D Platinum Software 5.0 (Amersham Bioscience, Geneva, Switzerland). Protein level of each spot was showed as the relative volume (vol%) of the spot in the gel. The average and standard derivations (SD) of DE protein spots were determined by three individuals of each group. *P* < 0.05 between different groups was considered statistical significantly using the independent *t*-tests.

### 2.5 Identification of protein by MALDI-TOF/TOF

As described in our previous paper [4], after digestion, peptide mixtures of protein spots were spotted onto the anchor chips, analyzed by the time-of-flight Ultraflex II mass spectrometer (Bruker Daltonics, Bremen, Germany). The mass spectrum acquired (*m*/*z* range: 700–4000; resolution: 15,000–20,000) was detected by Flex Analysis 2.4 and Biotools 3.0 software packages (Bruker Daltonics).

### 2.6 Bioinformatics analysis

As described in our previous paper [4], after the level of protein spot was standardized, Cluster 3.0 software was applied to analyze and cluster all DE proteins. Pathway Studio 5.00 (Ariadne Genomics, MD, USA) was applied to explore the relevant molecular events of all the DE proteins following 4t-OP exposure.

To further explore which posttranslational modification was the major modification impacted by 4t-OP, all DE proteins were matched and analyzed with the human sperm proteome 2.0 [2].
